# Building digital platform ecosystems through standardization: an institutional work approach

**DOI:** 10.1007/s12525-022-00552-0

**Published:** 2022-05-21

**Authors:** Carolina Costabile, Jon Iden, Bendik Bygstad

**Affiliations:** 1 Department of Strategy and Management, NHH Norwegian School of Economics, Helleveien 30, 5045 Bergen, Norway; 2 Department of Informatics, University of Oslo (UiO), Gaustadalléen 23B, N-0373 Oslo, Norway

**Keywords:** Digital platform ecosystems, Standardization, Standards, Longitudinal case study, O3, L15, M1

## Abstract

In this paper, we investigate the development of standards for technologies and work practices in a digital platform ecosystem. Standards are needed for technical and organizational compatibility across the actors’ different systems, technologies, data, and business processes. However, little is known about how actors achieve common standards in collaborative ecosystems where a clear platform leader is missing. Based on a longitudinal, qualitative case study of a digital platform ecosystem within the Norwegian aquaculture industry, we examined how the actors collaborated on building a digital platform ecosystem with the aim of fighting sea lice on salmon through standardization. We contribute to research and practice by providing a preliminary framework of four institutional work practices for standardization in digital ecosystems and three key lessons learned for guidance for practitioners.

## Introduction

Digital platforms have attracted increasing interest and have been approached from different perspectives, such as the market-oriented perspective and the technical perspective (Gawer, 2014). In this paper, we take an organizational lens and focus on digital platform ecosystems, considered open, evolving meta-organizations that coordinate actors through means other than a hierarchy (Gawer, 2014; Gulati et al., 2012; Jacobides et al., 2018). Digital platform ecosystems are often governed by a focal actor, such as Facebook, Apple, and Amazon, that controls the rules and interfaces with which the ecosystem’s actors must comply. Our focus is different as we examine collaborative digital platform ecosystems where independent companies in a business sector come together and, as a joint effort, develop and govern a platform and an ecosystem for mutual benefit. The development of these ecosystems faces a key challenge; the participating actors’ existing work practices and technological solutions are seldom harmonized. Consequently, for the ecosystem to succeed, standards must be developed and implemented, which is challenging in the absence of a clear platform leader (Miller & Toh, 2020).

As standards play a fundamental role in supporting the success of digital platforms (Wiegmann et al., 2017), it is timely and necessary to conduct research that contributes new knowledge about standardization in this context (Hanseth & Bygstad, 2015; Lyytinen & King, 2006; Tuczek et al., 2018; Wiegmann et al., 2017). Researchers have often overlooked the interwoven relationship between standard development and standard diffusion (Fukami & Shimizu, 2018), which is especially challenging in the absence of a focal actor that can enforce them. Moreover, focusing on standards as governing mechanisms may contribute to the discourse on whether digital platform ecosystems are emerging structures or whether they can be consciously designed (de Reuver et al., 2018). Based on this, we ask the following research question: *How can standards be developed for a digital platform ecosystem when there is no focal actor and where the actors’ existing technological solutions and work practices differ?*

To answer this question, we conducted a longitudinal, qualitative case study that followed the development of standards within a digital platform ecosystem in the Norwegian aquaculture industry. The aim of the ecosystem was to address the parasite sea lice, the industry’s most severe environmental challenge. This setting was relevant because of the heterogeneity in terms of technologies and work practices, the absence of a clear platform leader, and the actors’ previous opposition to cooperation. For our investigation, and specifically, to frame the actions involved in the standardization process, we rely on institutional work theory (Lawrence & Suddaby, 2006), and we consider standards as institutions that can be created through purposive actions (Lawrence & Suddaby, 2006; Lawrence et al., 2011). This theory sees agency as a distributed phenomenon, which we consider key for our case’s collective standardization effort, where the internal ecosystem’s members combine their skills and resources with other actors.

This study contributes to the literature on digital platform ecosystems and standardization, both theoretically and practically. Based on institutional work theory, we provide a preliminary framework for standardization in ecosystems without a focal owner and offer strategies and lessons learned for practitioners working in this area.

## Theoretical background

### Digital platform ecosystems

Originating within biology, the ecosystem perspective has shifted from focusing on competition among firms to coopetition, where actors jointly and simultaneously compete and cooperate (Hein et al., 2019). Ecosystems are perceived in different ways by different research streams (see Adner, 2017), but they can be defined as “an interdependent network of self-interested actors jointly creating value” (Bogers et al., 2019, p. 2).

Digital platform ecosystems are typical instantiations of ecosystems (Riasanow et al., 2021). Digital platform ecosystems are spreading widely and attracting considerable interest from practitioners and researchers within the fields of information systems, strategic management, economics, and marketing because these ecosystems change established business models in markets and industries (Asadullah et al., 2018; de Reuver et al., 2018; Hein et al., 2020).

Digital platforms have been approached from different perspectives. The market-oriented perspective—rooted within economics—has focused on two- or multi-sided platforms, where two or multiple groups of users are brought together (Bazarhanova et al., 2019; Otto & Jarke, 2019; Schreieck et al., 2016). The focus has been on network externalities and how the value of the platform on one side is dependent on the size of the other (Hein et al., 2020). The technical perspective considers digital platforms in terms of software and hardware as extensible codebases offering core functionalities that can be extended and supplemented through modular architecture and boundary resources, reaching economies of scale and scope (Asadullah et al., 2018; Hein et al., 2020; Schreieck et al., 2016). The focus is on co-creating value through the dynamics between the core functionalities and the developers’ capabilities rather than on enabling transactions among the different groups (Asadullah et al., 2018; Schreieck et al., 2016).

Although these perspectives are often considered separately, research may benefit from their integration (Gawer, 2014; Hein et al., 2020; Schreieck et al., 2016). With such an approach, digital platform ecosystems are evolving meta-organizations that coordinate actors, which can innovate and compete, and comprise technologies and associated work practices (Blaschke et al., 2019; Gawer, 2014; Schreieck et al., 2016). Thus, investigating how platforms integrate and govern an ecosystem of actors has become relevant (Hein et al., 2020).

Governing digital platform ecosystems is challenging due to the multiple different interests that must be balanced (de Reuver et al., 2018; Miller & Toh, 2020; Wiegmann et al., 2017). Governance has usually been referred to as the mechanisms that platform owners use to orchestrate their ecosystems (Halckenhaeusser et al., 2020; Schreieck et al., 2016; Tiwana et al., 2010, 2013). This research angle works best in traditional transaction-oriented platform ecosystems, where the platform owner establishes mechanisms (such as standards) to govern interactions within the ecosystem. However, the platform owner perspective is not suitable for illuminating the diverse platform landscape, where governance is increasingly a collective endeavor (de Reuver et al., 2018; Otto & Jarke, 2019). Investigating governance mechanisms for designing and building a digital platform ecosystem with distributed authority, decision making, and resource ownership is a challenging task that may benefit from a focus on boundary resources (de Reuver et al., 2018; Grant & Tan, 2013; Otto & Jarke, 2019; Schreieck et al., 2016). Boundary resources have been defined as resources that facilitate the interactions and the relationships between the actors (Ghazawneh & Henfridsson, 2013; Otto & Jarke, 2019) and are a useful angle from which to investigate patterns of interaction among the actors (Henfridsson & Bygstad, 2013). Various types of boundary resources have been suggested by the literature, including Application Programming Interfaces (APIs), Software Development Kits (SDKs), data, and standards.

### Standards and standardization

Standards are the result of a standardization process that aims at harmonizing entities such as technologies and work practices (de Vries, 1998). According to Brunsson et al. (2012), standards have four key characteristics. First, standards are explicitly formulated, and thus, they differ from implicit social norms. Second, standards regulate individual and collective behavior to achieve social order. Third, the decision to conform to standards is up to potential adopters. Standards’ regulatory power may depend not on the authority of a state but on the legitimacy and relevance that actors assign to them or on third-party pressure. Fourth, standards are meant for common use for a broad set of actors, even if, in some cases, groups of organizations, as consortia, may define standards applicable only to their own activities.

Standards have been classified in multiple ways. Without aiming for a comprehensive overview, we rely on the work of de Vries (1998) to highlight standard classifications. In relation to entities, standards can be categorized as basic standards or requiring standards. *Basic standards* offer structured descriptions of interrelated entities to facilitate human communication about these entities, such as terminology, classifications and/or codes, and descriptions of entity architecture. *Requiring standards* are a broad set that comprises, among others, quality standards (which set requirements to ensure a certain level of quality) and compatibility standards (which focus on the interrelation among entities).

Standards can also be classified according to their functions: intrinsic, extrinsic, and subjective (de Vries, 1998). Intrinsic functions refer to the description, record, and explanation of the agreed solutions to a certain problem. Extrinsic functions refer to the provision of transparency, interoperability, interchangeability, and information exchange. Subjective functions are related to specific actors’ interests, such as cost reduction and process facilitation.

Research considers the development of standards to be a dilemma that must be handled carefully (Fukami & Shimizu, 2018; Markus et al., 2006). Broad involvement is necessary but difficult to achieve, as standardization requires time and resources (Markus et al., 2006; Van de Kaa et al., 2015; Zhao et al., 2011). However, too many participants may slow down the process or make the standard too complex. Moreover, the heterogeneity of the stakeholders’ interests may hamper the speed of standardization, but if the interests of those involved are not sufficiently represented, the standard may not be adequately developed or diffused (Markus et al., 2006; Zhao et al., 2011). Standard development and standard diffusion are failure-prone processes, and research suggests that solutions which address the former may fail to address the latter. However, researchers often overlook the interwoven relationship between standard development and diffusion (Fukami & Shimizu, 2018), an especially relevant issue for a digital platform ecosystem without a focal actor.

### Institutional work

To examine how standards were developed in the present case, we use *institutional work theory* as the theoretical lens, a theory originating in the seminal work of Lawrence and Suddaby (2006). Institutions are fundamental elements of social life that affect individual and collective beliefs and behavior, and institutional work is used to examine purposive actions aimed at creating, maintaining, and disrupting institutions (Lawrence et al., 2011). Work is seen as a physical or mental effort to reach a goal; it is characterized by a future-oriented intentionality with the strategic aim of reshaping institutions (Lawrence et al., 2011).

Compared to an institutional perspective focused on the macrodynamic (i.e., the processes that lead to large-scale social and economic change), institutional work is concerned with the lived experiences of individuals and organizations, and their link to the institutions that shape and are shaped by them (Lawrence et al., 2011). Agency is not confined to institutional entrepreneurs with considerable resources and skills. Instead, a distributed perspective is adopted by including a wider set of actors that support and facilitate the creation of institutions (Lawrence & Suddaby, 2006; Lawrence et al., 2011).

For our analysis, we draw on the seminal work of Lawrence and Suddaby (2006), in which the authors provide examples of *practices* that actors can purposely use to create institutions. Actors *construct identities* (i.e., reconfigure group beliefs), which can come from within or outside the group and are often linked to the development of professional identities. Regarding this practice, Oakes et al. (1998), cited by Lawrence and Suddaby (2006), examined how the government department responsible for museums, by introducing business planning, encouraged museum personnel to see themselves as business workers and entrepreneurs who had more agency and could take more risks instead of as only researchers, educators, or curators. Further, actors *construct normative networks*, that is, interorganizational connections that can be established alongside extant institutional arrangements and that can mimic or simply supplement and support the state’s regulatory activities. These networks can represent the relevant peer group with respect to which practices can be sanctioned or judged as compliant. Guler et al. (2002) explained how ISO 9000 practices were diffused through the promotion and network established by engineers and production managers.

Moreover, actors *educate* to provide skills and knowledge to support the creation of the new institution. This is usually done by large dominant actors but can also be conducted by marginal actors acting collectively. An example is the institutionalization of recycling programs at American universities, which was achieved by educating a large student population through workshops, guidelines for action, and access to success stories at other universities. Another cognitive type of institutional work is *mimicry*, which leverages extant taken-for-granted practices, technologies, and rules with which to associate new practices, legitimate them, and ease their adoption. For instance, to institutionalize electric light, Edison designed the bulbs to be indistinguishable from the familiar existing gas systems and kept the wattage aligned with that of gas bulbs (even if bulbs could have produced more light). Actors can *advocate* to acquire legitimacy through trustworthy and relevant resources and agents. It can be valuable for marginal actors to be able to effect new institutions; and creating cognitive legitimacy for the new institution can take several forms, such as lobbying, advertising, litigating, and coercing. For example, Holm (1995) showed how the close relationship between the Fisherman’s Association and the Labor Party helped preserve fishermen’s interests in Norway’s Herring Act. In this study, we used institutional work as a theoretical lens to frame the practices for standardization that we recognized in the analysis of our case.

## Methodology

To address the research question, we followed an in-depth, longitudinal, qualitative case study approach. Case studies are considered appropriate for understanding complex social phenomena (Yin, 2014) and topics on which research and theory are in their early stages (Benbasat et al., 1987). We investigated the development of a digital platform ecosystem within the Norwegian aquaculture industry. We selected this platform for several reasons. First, it operates within a traditional industry, where the actors have a long history. Second, the case involves heterogeneous actors, practices, interests, data, and technologies. Third, actors have previously shown resistance to sharing data and their internal practices. Finally, the platform does not have a leader; governance is shared among ecosystem members.

### The setting

The selected case platform began operating in 2017 to address the parasite sea lice, the industry’s most severe environmental challenge. Because sea lice spread very quickly and can easily affect adjacent farming companies, joint efforts and data sharing were considered fundamental to prevent outbreaks. Based on data from farmers’ cages collected through different technologies (such as sensors and cameras) and through big data analytics, algorithms, and artificial intelligence, the central platform creates two-week sea lice forecasts. In the beginning, the data were manually entered into the platform; later, they were pulled automatically through APIs. The core platform is managed by a technical partner, whereas the entire ecosystem is facilitated—but not controlled—by an innovation cluster consisting of a set of partners and members collaborating and sharing knowledge. The ecosystem’s governance is shared among its members.

In 2019, the ecosystem’s members acknowledged that the data quality was not good enough. This lack of quality had a negative impact on forecast trustworthiness, and thus, on achieving the sustainability goal. Therefore, the need for standardization emerged. The scope of standardization embraces technologies and work practices, comprising architecture, compatibility, quality, and terminology standards (de Vries, 1998), as shown in Table 1.

**Table 1:** Standardization workflows within the case

Standardization workflow	Standards (to be) developed	Reasons why it was considered important	Type of standards and function (de Vries, 1998)
Sensor data	Sensor infrastructure standard for sea farms below the water based on the Open Platform Communications (OPC) Unified Architecture	Data across facilities were not comparable. The data were stored in proprietary systems, and their value was not fully realized. Different technologies and systems could not be integrated	Architecture (basic standards), Compatibility (requiring standards) Enabling interoperability and interchangeability
Environmental data	Practices and methods related to measurements taken inside the pens	Data comparability was not possible, because the data were collected differently across farmers, at different times and positions in the cage, with different equipment	Quality (requiring standards) Enabling performance
Fish health data	Definitions, meanings, classifications, and industry language	Different classifications (e.g., of causes of fish death) made it difficult to make proper operational decisions and to respond to the pressures of authorities and researchers	Terminology and classification and/or codes (basic standards) Providing transparency

**Fig. 1:**
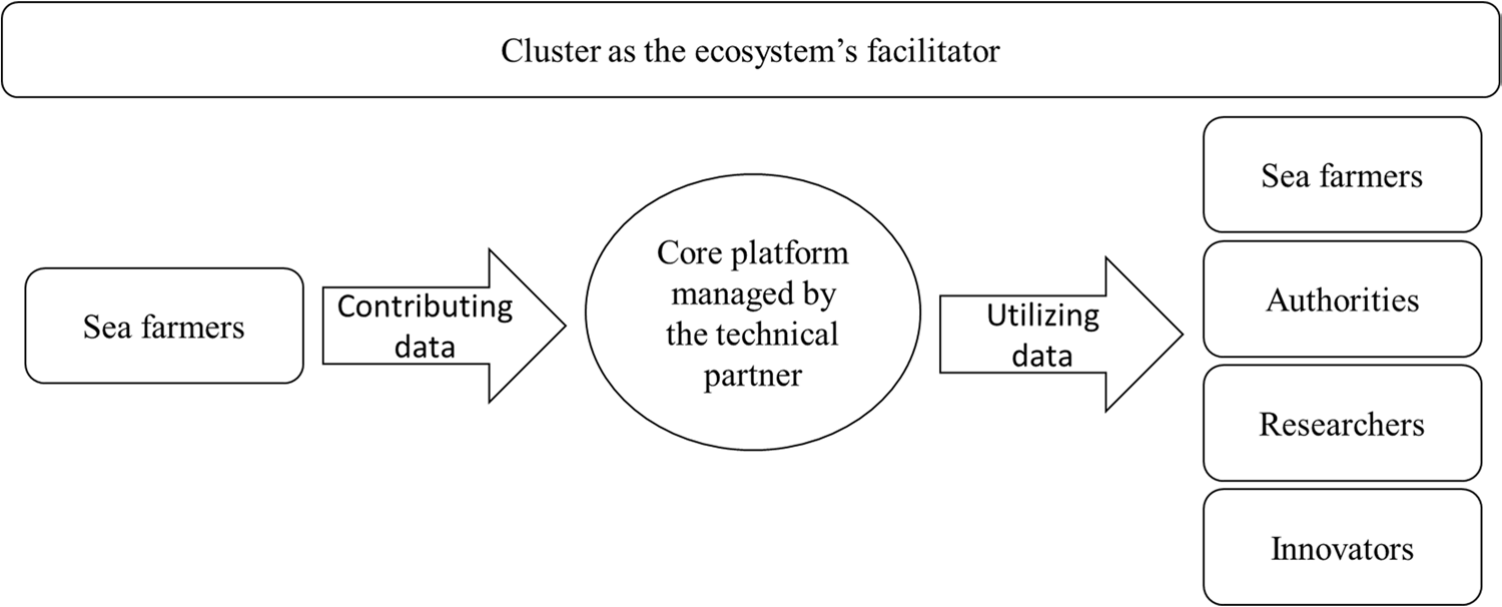
The ecosystem’s actor groups

With the experience gained and the interest that external actors had begun to show in the data generated by the ecosystem, the members understood that the platform could develop into a hub for the entire industry. Government authorities could benefit from a better understanding of the industry’s status to align policies and regulations. Research institutes could benefit from quality data for their studies. Service and product innovators could benefit from developing new services (e.g., automatic sea lice counting). Figure 1 provides an overview of the ecosystem’s actors.

Looking at digital platform ecosystems as evolving meta-organizations (Gawer, 2014), the case moves from facilitating interaction within a single group of users (i.e., sea farmers) to enabling interaction across multiple groups (i.e., sea farmers, authorities, researchers, and innovators; Staykova & Damsgaard, 2015). In this setting, standards represent a governance mechanism that can subsidize both sides.

### Data collection

We collected data through semi-structured interviews, documents provided by informants, online articles, and participation in a professional aquaculture industry course. Interviews were the main data source. We conducted 19 interviews from fall 2019 until spring 2021, divided into two rounds. In the first round, we focused on the launch and context of the digital platform ecosystem and what led to the need for standardization. In the second round, we focused on the standardization process. Questions in the first round concerned the actors’ roles, their reasons for and perspectives on their involvement in the ecosystem, technologies and organizational solutions, their evolution over time, and the challenges that led to standardization. In the second round, questions concerned the standardization process, how it was structured, the actors involved, and their actions.

We followed purposeful sampling (Marshall, 1996), interviewing actors who could provide us with key and useful information because of their involvement or interest in the standardization work. We interviewed actors with different roles (e.g., technical development personnel, senior innovation managers, and researchers) in different companies (e.g., the cluster, sea farms, research institutes, and the technical partner) to secure a variety of viewpoints. Some respondents were interviewed more than once. An overview of the informants is provided in Table 2.

**Table 2:** Informants

Informant	Organization	Role	Comment
1	Sea Farm A	Project Manager, Leader – Sensor Data Standardization	Interviewed twice
2	Sea Farm B	IT Business Partner Feed and Farming	
3	Sea Farm C	Digitalization Director	Interviewed twice
4	Cluster	Innovation Manager, Leader – Environmental Data Standardization	Interviewed three times
5	Technical Partner	Project Manager	Interviewed twice
6	Sea Farm C	Chief Technical Officer	
7	Cluster	Ecosystem’s Project Manager	
8	Innovation Center	Senior Project Manager Innovation	
9	Cluster	Chairman	
10	Consulting company	Project Leader – Fish Health Data Standardization	
11	Supplier	Solution Manager	
12	Sea Farm A	Technical Development Manager/Head of R&D on Sea Farms	
13	Research Institute	Senior Researcher	
14	Sea Farm A	Head of IT & Systems	

The interviews lasted between 30 and 90 minutes and were based on an interview guide, which left room for the exploration of new areas. Most of the interviews were conducted and recorded digitally due to the Covid-19 pandemic and transcribed verbatim. Using documentation from informants and online archival data, we crosschecked the interview data and collected relevant contextual information about the case and the aquaculture industry. On this last point, the first author was involved in a professional sustainability and digitalization course within the industry.

### Data analysis

The data analysis was conducted in six steps, as shown in Table 3.

**Table 3:** Data analysis steps

Step	Activity	Output
1	Reading transcripts, notes, and documents	Familiarization with the data and the case
2	Analyzing the chronological development of standards	Written narratives of each standardization workflow
3	Coding data for each workflow separately	Identification of actors and their individual activities for standardization
4	Comparing the different workflows; creating a visual timeline of the standardization process	Identification of practices used to achieve common standards
5	Abductively analyzing the case through the empirical data and the theoretical lens	Framing of the case’s practices used in the standardization work
6	Comparing and reconciling researchers’ individual interpretations	Agreement on the case’s interpretation

First, we independently read transcripts, notes, and documents several times to familiarize ourselves with the data. Second, together, we created a chronology of each standardization workflow by writing narratives, which served as a data organization device for further analysis (Langley, 1999). Third, we independently coded the data to identify key aspects of the standards under development (i.e., the actors involved, how the work was organized, their strategies, and the challenges along the way). Fourth, we created a visual timeline of the overall standardization work and compared the different workflows to derive practices to use to achieve common standards. Fifth, through an abductive approach (Dubois & Gadde, 2002), we independently moved back and forth between the case analysis and the theoretical lens (i.e., institutional work theory; Lawrence & Suddaby, 2006) to frame the practices used by informants in the standardization work. Sixth, we compared and reconciled our individual interpretations. In the next section, we present the findings according to the theoretical lens.

## Findings: standardization work

We structure the findings in terms of the four institutional work practices that we uncovered in this case.

### Constructing identities

The Norwegian aquaculture industry has long been characterized by an uncontrollable and inaccessible flow of sensor data stored in closed, proprietary systems, which makes it difficult to share data within the ecosystem. Even within a single company, data from different facilities can be in different formats. Moreover, data quality was not assured because the data were without context. Some data were captured by sensors, while others were captured manually. In addition, the data were stored in proprietary formats determined by the individual vendor, making it impossible to combine or compare data from systems from different vendors.

This situation with proprietary and incompatible systems was well summarized by the digitalization director of Sea Farm C: “The history here in our industry is that there have been two big Norwegian vendors of management systems that had their own proprietary platforms that kind of locked you in as soon as you selected one of those players.”

As part of an ecosystem aimed at fighting sea lice, the farmers became more aware of the locked-in situation and its consequences, and they decided to take a more active role in developing technology. Because they were big and valuable customers, they understood that they should use their buying power to require open standards and system interfaces. Moreover, given the increasing interest in the data generated by the ecosystem, the farmers understood that through standardization, they could move toward a knowledge-based industry and get more attention:

It is tremendously easier to duplicate the fact-based knowledge than the experience-based knowledge (…) And it is also much easier to kind of nurture the curiosity culture that we actually want to foster in the industry as such because you can nurture the curiosity by facts. (Digitalization Director, Sea Farm C)

Through this realization, the sea farmers were socially constructing a new identity and reconfiguring their beliefs: The farmers became aware that together, and with the cluster, they could reverse the power relationship to access more and better-quality data for a more sustainable environment. To this end, the farmers decided to initiate a standardization program following an open, voluntary approach.

### Constructing normative networks

To build the standards within an industry-wide collaboration of interested actors and to access a broad set of competences, the ecosystem’s members sent out an invitation in a post on the cluster’s website and followed up with newsletters. Although participation was voluntary and based on competence and capacity for the sensor standardization workflow, within two weeks, 47 actors had signed up, and more participated in the kick-off event that followed.

However, despite the anticipated benefits that standardization could bring, at first farmers were reluctant to share sensitive information and their in-house practices with their competitors. This “fear of sharing” contradicted the principle of openness, which meant that any input and suggestions for the standards should be publicly available and openly scrutinized. A senior researcher explained:

This openness is very hard for aquaculture. The aquaculture industry doesn’t want to share data (…) And they (the sea farmers) don’t want to necessarily reveal the inner working of what they do. In the first meeting I was in, there was a discussion about this, about the fact that, in some cases, you might be revealing much more than you want to your competitors.

To overcome this resistance, meetings and leveraging previous working relationships were key. In addition to the farmers, and to secure an industry-wide engagement, the two dominant software vendors of management systems were brought onboard. This involvement was also important in preparing them for the work they needed to do to implement the standards in their systems. Moreover, for one of the standardization workflows, the recruited project manager had previously worked for one of the two software vendors.

Overall, the informants explained that for this industry-wide collaboration to succeed, shared ownership was fundamental. Shared ownership meant that each actor could influence the direction of the standards under development and was considered crucial for diffusing and implementing the standards. Shared ownership was achieved through the way standardization was organized. Each workflow (see Table 1) was organized with one working group and one reference group. The working group was responsible for writing a first-draft document for the standard. This draft document was then sent “on hearing” to the reference group for feedback. If there were comments and suggestions, a new draft document was developed. This cycle was repeated until consensus was reached. The final standard was not influenced by the actors’ size or power in the industry but was based on value, competences, and supporting arguments.

### Educating

To further smooth the standardization work, the ecosystem’s members provided all the actors involved with knowledge and mutual understanding for developing common standards. In addition to meetings to handle feedback on drafts sent on hearing, other meetings and webinars were organized to nurture a broad interest in the ecosystem, what standardization could bring and solve, and the consequences of not standardizing.

For instance, the ecosystem’s steering committee considered it important to align the farmers’ different perceptions of standardization and the way it was (more or less) prioritized across them. “If they don’t have the same priority for this, then it’s even more difficult to achieve what you need. Communication is key to find a common priority, a balance” (Head of IT & Systems, Sea Farm A). Meetings were also important to shape a positive attitude toward standardization, which is often considered to limit freedom for innovation. People’s attitudes, more than the use of new technologies, were perceived as critical. The digitalization director, Sea Farm C, clarified:

And that’s the biggest change in digitalization. It is not the technology that is the problem; it is the people. That is step number one, but this is actually the hardest one (…) So we have to put efforts into that so that they see the benefits for themselves and for the industry.

Beyond communication and transparency to enhance standards development, the ecosystem’s members were taking steps to foster the subsequent diffusion and acceptance of the standards. Members were aware of the importance of developing user guidelines to assist practically in implementing the standards. Moreover, the farming companies organized internal training to align work practices with the new standards. Overall, educating was key for developing and diffusing the standards, which our informants described as “very much connected” (Senior Researcher, Research Institute).

### Mimicry and advocacy

Standardization was not new in the aquaculture industry, but previous attempts failed for several reasons due to low technological maturity and poor standards. However, it was understood that previous work could be leveraged and revised. There was no need to reinvent the wheel; instead, the ecosystem’s members worked on coordinating existing standards and putting them into a system. Specifically, some aquaculture standards developed in 2012 by the national standardization body were considered the starting point, and they were revised under the auspices of the national body.

The project leader for the fish health data workflow clarified:

We are participating in an industry project with the entity called Standard Norway making different kinds of standards for different industries, and in aquaculture, there are several standards, but one of these standards is called NS9417 that is a standard for (…) definitions used, and special names and processes, and definitions used in the industry, a kind of industry language. And we also work with the seafood association. So, it is important to get involved with different stakeholders in the industry, like fish health services, laboratories.

Moreover, the standardization work leveraged other industries’ knowledge and practices. For instance, with the aim of defining codes related to fish health and causes of death, the project leader for the fish health data workflow stated:

For aquaculture, it is important to obtain standards from outside, used in other industries (…) In the fish health workflow, we have looked at agriculture and animal husbandry, what kinds of classifications for diseases and causes of death exist. And in medicine, you have this (…) international standard of classification of causes of death of people. In our project, we kept an eye on this because there is no need for us to start from zero.

Involving external experts was considered key in creating high-quality standards. For instance, some of the business and academic people involved in the standardization work in 2012 were engaged. Moreover, in the sensor data workflow, involving biologists allowed for useful add-ons to the technology. A senior researcher who was invited to participate in the sensor standardization explained, “I have worked towards adding other elements such as light, better light quality data because (…) light is the biggest driving force of biology. I mean, it is more important than temperature.”

Another example is that, for the fish health standardization workflow, most work was conducted by employees at the Veterinary Institute and the Norwegian University of Life Sciences:

Inside that group, there are people educated in the fish health science, but also people working as fish health professionals or managers in fish farms, so they have the practical experience, and some have been 40–50 years in the industry both in the academics and out in the field. So, they know very well the needs, and they also have experience from animal husbandry and also from fish farms, fish health services for many years (…) So, they also have the trust. (Project Leader for fish health data standardization workflow, Consulting Company)

Overall, existing standards, broad involvement, and participation among the ecosystem’s members, together with knowledge from academics and experts in other industries, were utilized and combined to develop the standards. This approach not only helped legitimize the standards but also contributed to their implementation and use. Table 4 provides an overview of the different institutional works that were put in place to jointly build and diffuse industry-wide standards in an ecosystem where there is no focal actor.

**Table 4:** Overview of the institutional works

Institutional work	Importance in the standardization work	Issues raised	Actors	Outcome
Constructing identities	Preparatory work – Standard development	Low data quality, availability, and comparability	Internal ecosystem’s members	Reconfigured beliefs, awareness of possibility of reverting power relationship with dominant software vendors
Constructing normative networks	Standard development and diffusion	Open standards can benefit the whole industry and allow for integration of the systems	Internal ecosystem’s members and researchers, software vendors, other farmers	A standardization network where all industry actors could participate and influence the standards under development
Educating	Standard development and diffusion	Different perceptions and attitudes about the value of standardization	Ecosystem’ steering committee	A common and positive attitude toward the standards
Mimicry and Advocacy	Standard development and diffusion	Increase legitimacy and quality of standards	Internal ecosystem’s members and national standardization body, University of Life Sciences, and Veterinary Institute	New standards based on extant standards and knowledge

## Discussion

This study was guided by the research question, *how can standards be developed for a digital platform ecosystem when there is no focal actor and where the actors’ existing technological solutions and work practices differ?* To answer this question, we investigated a platform ecosystem where multiple companies came together to solve a common problem that they understood could not have been solved by each actor alone. The short answer is that standardization is a gradual consensus process, encompassing four institutional work practices.

### Standardization in collaborative platform ecosystems

By looking at standards as a product of institutional work (Lawrence & Suddaby, 2006), this study contributes by uncovering the practices involved in standardization. We found that standardization is a dynamic process with activities influencing beliefs, values, understanding, and the roles of those involved. Moreover, in line with the work of Slager et al. (2012), this study shows how a broad set of internal and external actors can combine their experience, competences, and skills to move the standardization process forward. *Constructing identities* allowed a group of actors of the same type (i.e., sea farmers) to acknowledge their role and joint power in initiating a standardization process and challenging the status quo, characterized by a lock-in in the vendors’ proprietary systems. Farmers understood that only together could they increase compatibility among and across their own facilities and attain improvement to achieve their sustainability goal. *Constructing a normative network* was relevant for creating a collaboration that spanned multiple groups of actors. In this case, the initiative was made public through the cluster’s website, newsletters, and events and was open to anyone who wanted to contribute. Shared ownership was key in creating an environment in which any actor, despite its size, could have a say in and a voting right to influence the direction of the standards in the making. Actors with divergent interests, such as dominant software vendors, were not excluded; instead, their engagement was considered pivotal from the very beginning. Overall, shared ownership and engagement allowed not only to create an arena for collaboration but also to make the standards easier to subsequently accept due to participation in the development phase. This is in line with existing research that has shown IT vendors’ contribution benefits users in ensuring that the standards under development will be technically feasible (Zhao et al., 2011). Farmers’ participation also ensures that resources spent by software vendors in adjusting their software and technologies are not wasted. *Educating* allowed for building mutual knowledge and understanding of standardization to reduce divergences in terms of priorities or perceptions. Moreover, this institutional work also aimed at smoothing the adoption of the standards under development by working on user guidelines and arranging internal training. *Mimicry and advocacy* considered existing standards, including those in other industries, as valuable sources on which to build standards for the aquaculture industry; this institutional work also emphasized the importance of relying on trustworthy and authoritative actors (e.g., experts and the official standardization body) that could increase the legitimacy of the standards under development.

Our analysis suggests that the development and diffusion of standards are highly intertwined, a relationship that has often been overlooked in the literature (Fukami & Shimizu, 2018). Standard diffusion was addressed from the beginning, and attempts to deal jointly with development and diffusion were put into practice, such as relying on shared ownership, fostering a common understanding, and engaging trustworthy actors. This finding confirms the findings of Markus et al. (2006) and is different from most research (as described in Markus et al., 2006) that usually suggests different solutions to tackle the two processes individually. Moreover, this case stresses the relevance of broad involvement, contrasting the regulated actor approach that research on the consortia mode has considered successful (e.g., Weiss & Cargill, 1992 in Markus et al., 2006). We argue that inclusiveness, rather than exclusiveness, may promote standardization.

Furthermore, involving a broad set of actors in creating the standards increased acceptance of them. Participants may also become advocates, pushing future suppliers and customers to adopt the standards (Boh et al., 2007). In this way, the ecosystem will be able to scale up with more and different user groups that can easily join and build value based on the data provided.

This study suggests that, in addition to compatibility standards, additional types, such as quality, terminology, and classification standards, are also relevant for the development of ecosystems. These standard types fit the sociotechnical features of platform ecosystems. In studying standardization, we recommend a shift from seeing it as a pure technical study object and discourse to a more comprehensive one. This comprehensive approach is in line with the fact that standards are growing rapidly in variety (Hanseth & Bygstad, 2015). As previously suggested by Nickerson and Muehlen (2006), there is a need for a focus on ecologies of standards instead of individual ones.

### Implications for practice

The implications for practice can be summarized in the following three key lessons learned.

#### Engage and inspire a broad set of key actors

This case shows that it is necessary for members of a digital platform ecosystem without a dominant player to collaborate broadly within the industry. An open approach will give the ecosystem access to a broad and diverse set of external competences and skills. Collaborating with representatives from various external stakeholders (including software suppliers) increases the success rate of development and subsequent diffusion (Markus et al., 2006; Zhao et al., 2011). Involving a broad set of actors and key players may strengthen the perception that standards have been developed by accounting for costs and impacts on all relevant actors (Boh et al., 2007). This has a strong influence on the standards’ legitimacy, which can be augmented through mobilization of political and regulatory support (Lawrence & Suddaby, 2006). As shown in this case, the ecosystem’s members worked under the auspices of influential external actors (i.e., national standardization bodies and academic institutions). In ecosystems lacking a focal, dominant actor, engaging with external actors with knowledge and authority will increase the legitimacy of the standardization process and the standards (Lawrence & Suddaby, 2006), which will enhance the subsequent adoption and increase the ecosystem’s value.

#### Leverage extant standards and knowledge

Developing standards does not have to come from a tabula rasa approach. This case shows that revising extant industry standards and aligning them with the current business scenario can be a viable approach. This approach has also proved to be successful in previous standardization works. For example, the Norwegian health sector followed a pragmatic approach by first making use of available standards and then modifying them when necessary (Hanseth et al., 2012). The present case also shows that it may be a good strategy to use knowledge matured in other industries and to leverage actors (individuals and organizations) with experience in previous standardization processes. Grafting (i.e., defining standards based on extant standards to improve their functionality and usefulness) and extension (i.e., adding new elements to extant standards) represent useful strategies for changing and revitalizing previous standardization attempts (Egyedi & Blind, 2008).

#### Develop standards with diffusion in mind

This study makes it evident that standard development and standard diffusion are highly interwoven. The development phase should be managed with the subsequent implementation phase in mind. An inclusive, transparent, and open approach in the development phase may shape a positive attitude among the ecosystem’s members toward implementing the standards in their technologies and work practices. Shared ownership was found to be a pivotal element in ensuring the ecosystem members’ acceptance because it is easier to accept, conform to, and advocate for using standards one has contributed to (Boh et al., 2007). As standards play such an important role in the success of digital platform ecosystems (Wiegmann et al., 2017), their acceptance can make a difference regarding the ecosystems’ development and reputation, especially for ecosystems without a focal actor.

#### A preliminary framework for standardization in ecosystems

**Fig. 2:**
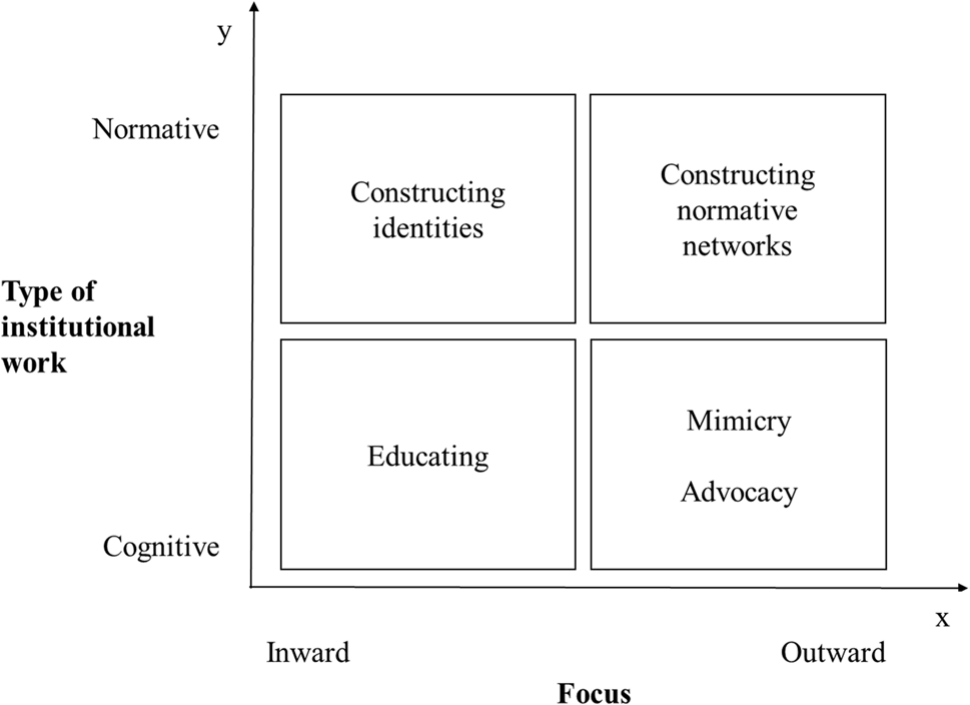
A preliminary framework for standardization in digital platform ecosystems

Based on our analysis and institutional work theory, we propose the following preliminary framework for standardization within digital platform ecosystems, as shown in Fig. 2.

The proposed framework has two dimensions. On the x-axis is the focus that the standardization process can adopt, inward-looking or outward-looking (including external stakeholders). On the y-axis are the institutional work practices that can act as normative (i.e., on beliefs, values, and roles) or cognitive (i.e., on meanings).

The framework suggests that to standardize, an ecosystem can leverage well-known institutional work practices starting with the preparatory work of *constructing identities*, serving as a tool for reconfiguring the beliefs of the ecosystem’s current members, with the aim of building shared awareness (Lawrence & Suddaby, 2006). This is in line with previous research that has acknowledged users’ coalitions as a means of ensuring users’ involvement in standardization efforts (Foray, 1994 in Markus et al., 2006). Then, actors can leverage the three other institutional work practices. The outward-looking practice of *constructing normative networks* allows external actors to be involved in standardization, whereas the inward-looking practice of *educating* ensures shared knowledge and understanding, thus building up support for standard development. *Mimicking* available standards and knowledge in the industry (outward focus) provides a baseline for exploring new possibilities (Lawrence & Suddaby, 2006). Mimicry legitimizes the new practices, whereas advocacy helps marginal actors shape cognitive legitimacy for participating in standardization (Lawrence & Suddaby, 2006). Although there is no unique way to develop standards (Biddle, 2016), we argue that a standardization process involving the four institutional work practices in an iterative way is appropriate for designing a digital platform ecosystem with no focal owner.

#### Limitations and further research

Standardization in digital ecosystems is an emerging field, and although many insights from standardization research are valid, some aspects of digital ecosystems present new theoretical and practical challenges. One is the question of how standardization in such regional ecosystems, as presented in this study, can be scaled up to encompass an entire sector and interconnect with other ecosystems. This issue may be investigated within an industry with several parallel platform ecosystems to gain insight into the strategies for merging or combining them at the industry level. Another issue is how the “non-generic complementarity” (Jacobides et al., 2018) of digital ecosystems affects standardization. It could be worth investigating whether non-generic complementarities may smooth collaboration and coordination in developing standards and whether they may reduce or increase the relevance of some of the institutional work practices that we identified in our case. Finally, we call for additional empirical research to validate and further enhance the preliminary framework.

## Conclusion

This study investigates standardization within a collaborative digital platform ecosystem. Building on institutional work theory and our analysis, we envisage that standardization is a dynamic and gradual consensus process based on four institutional work practices that address standard development and diffusion. We organized these practices in a preliminary framework. We also provide three key lessons learned for practitioners involved in standardization for collaborative digital platform ecosystems.
